# Gene Co-Expression Network Analysis Associated with Endometrial Cancer Tumorigenesis and Survival Outcomes

**DOI:** 10.3390/ijms252212356

**Published:** 2024-11-18

**Authors:** Alexis J. Clark, Rajesh Singh, Regina L. Leonis, Eric A. Stahlberg, Zachary S. Clark, James W. Lillard

**Affiliations:** 1 Department of Microbiology, Biochemistry and Immunology, Morehouse School of Medicine, Atlanta, GA 30310, USA; ajclark@msm.edu (A.J.C.); rsingh@msm.edu (R.S.); 2 Department of Obstetrics & Gynecology, Morehouse School of Medicine, Atlanta, GA 30310, USA; rleonis@msm.edu; 3 Cancer Data Science Initiatives, Frederick National Laboratory for Cancer Research, Frederick, MD 21701, USA; eric.stahlberg@nih.gov; 4 Department of Information Technology, Clayton State University, Morrow, GA 30260, USA; zclark4@student.clayton.edu

**Keywords:** multi-omics, prognostic markers, precision oncology, endometrial cancer

## Abstract

Endometrial cancer (EC) presents a substantial health challenge, with increasing incidence and mortality rates. Despite advances in diagnosis and treatment, understanding the molecular underpinnings of EC progression remains unknown. In this study, we conducted a comprehensive investigation utilizing The Cancer Genome Atlas (TCGA-UCEC *n* = 588) data to analyze gene co-expression patterns, elucidate biological process pathways, and identify potential prognostic and diagnostic biomarkers for EC, using weighted gene co-expression network analysis (WGCNA), differential gene expression, survival analysis, and functional analysis, respectively. We determined that the Green module (M5) was significantly correlated with patient survival. Functional analysis of the genes in module M5 indicates involvement in cell cycle regulation, mitotic spindle assembly, and intercellular signaling. *TPX2*, *BUB1*, and *ESPL1* were among the top differentially expressed genes in the Green module, suggesting their involvement in critical pathways that contribute to disease progression and patient survival outcomes. The biological and clinical assessments of our findings provide an understanding of the molecular landscape of EC and identified several potential prognostic markers for patient risk stratification and treatment selection.

## 1. Introduction

Endometrial cancer (EC) represents a significant health burden worldwide, with rising incidence and mortality rates observed in recent years [1]. This increase is particularly alarming due to the health disparities in EC incidence and mortality rates across different racial and ethnic groups. Statistics have shown that from 2009 to 2018, non-Hispanic White women had a lower incidence rate (~1.2% increase) compared to the 3.5% increased incidence in African American women [2,3]. Disturbingly, African American women also experience double the mortality rate from EC [4]. Several factors contribute to the increased incidence of EC, including obesity and decreased fertility [5]. Despite advances in diagnosis and treatment, a comprehensive understanding of the molecular mechanisms driving EC progression remains elusive. Identifying biomarkers associated with disease development and prognosis is crucial for improving patient outcomes through early detection, personalized treatment strategies, and accurate prognostic assessment.

The Cancer Genome Atlas (TCGA) project has played a pivotal role by generating large-scale genomic, transcriptomic, and clinical data for several cancer types, including EC. The TCGA project employed whole-genome sequencing, exome sequencing, MSI assay, copy number analysis, and proteomics to classify ECs into four genomic marker subtypes: ultramutated/polymerase (POLE-mutated), hypermutated/MSI(MSI-H), copy number-low (microsatellite stable), and copy number-high [6]. This molecular characterization has paved the way for the development of targeted therapies, which have shown promise in improving patient outcomes. For example, EC with ultramutated or MSI harbor a high prevalence of somatic mutations, making them candidates for immunotherapy [7]. Immunotherapy was first incorporated into the treatment of recurrent endometrial cancers with microsatellite instability [8], and is now approved for both recurrent and front-line settings of advanced stage EC, regardless of biomarker status [8,9,10].

In addition to immunotherapy, other targeted therapies have emerged based on specific molecular alterations in EC. HER2 inhibitors, like trastuzumab, are used in combination with chemotherapy for aggressive EC that overexpresses HER2 [11]. Poly ADP-ribose polymerase (PARP) inhibitors, like Olaparib and rucaparib, block the enzymes involved in repairing damaged DNA, making them effective in cancers with dysfunctional DNA repair pathways and BRCA mutations. mTOR inhibitors, like Everolimus, target mTOR, a protein that regulates cell growth, division, and survival. The identification of biomarkers and significant biological pathways is crucial in driving clinical drug development and targeted therapies.

EC is a heterogeneous disease with distinct molecular profiles that may contribute to challenging diagnostic and treatment strategies. Identifying prognostic genes can improve personalized therapies and patient outcomes. The goal of this study is to identify gene co-expression modules associated with survival outcomes in EC. We aim to use computational approaches on TCGA data to investigate the molecular landscape of EC to support prognostic biomarker discovery for the development of future targeted therapies. TCGA data are important for our study because of their clinical annotations, which allow for survival analysis and WGCNA clinical associations.

In this study, we employed weighted gene co-expression network analysis (WGCNA) to identify genes associated with EC patient vital status. WGCNA allows for the identification of gene modules in association with expression patterns. These modules are frequently functionally related, which provides insight into regulatory networks and key biological processes that drive the disease. This method allowed us to prioritize genes that may serve as biomarkers due to their role in the co-expressed networks. Additionally, we conducted differential gene expression analysis to reveal the expression patterns of the hub genes in tumors versus normal tissue. We also conducted a survival analysis to test the significance of these DEGs in patient survival outcomes. Through this comprehensive analysis, we identified novel biomarkers that reflect tumor biology and have prognostic potential. These findings enhance our understanding of the molecular mechanisms driving EC progression and may contribute to improved patient outcomes by providing new targets for personalized therapeutic strategies.

## 2. Results

### 2.1. WGCNA

**Figure 1:**
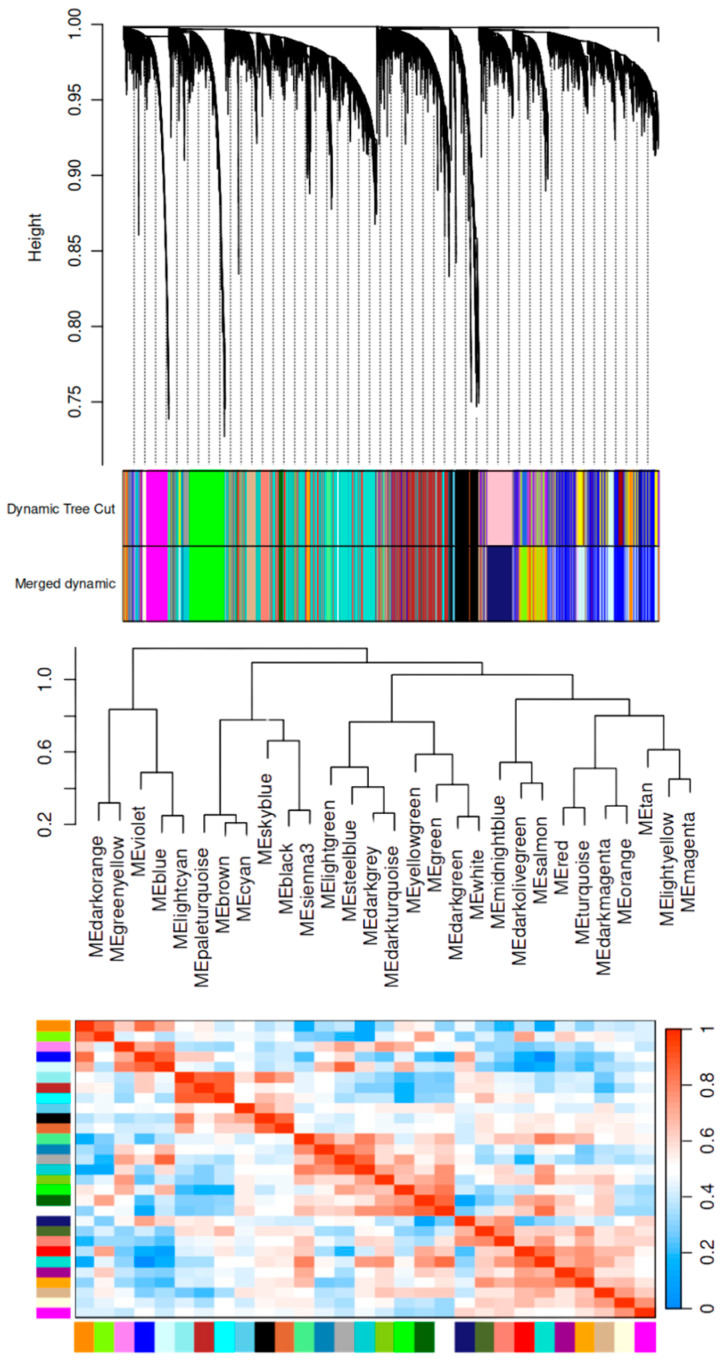
Cluster dendrogram and module–trait relatedness of the expressed genes based upon the topological overlap with the corresponding module colors. The top panel represents the gene dendrogram obtained by hierarchical clustering the gene expression similarity based on consensus topological overlap, with the corresponding module colors indicated by the color row. Each leaf (end) of the dendrogram represents a gene; the branches connect genes that are more similar in expression. The height of the branches represents the dissimilarity between clusters of genes. The higher the branch point, the more dissimilar the gene clusters are. The dynamic tree cut color row represents the initial module assignments based on the dynamic tree cut method. The second color bar shows the module assignments after merging similar modules based on their eigengenes. The difference in the number of modules (colors) indicates that several smaller modules were merged, making more statistically and biologically robust results. The module–trait relatedness clustering visualizes how related each module is to one another.

We performed weighted gene co-expression network analysis (WGCNA) on 14,802 genes among 588 patient samples to investigate the co-expression patterns and their association with clinical traits. The patient-matched clinical samples were obtained from TCGA. Our WGCNA analysis identified 25 module eigengenes (MEs), which are the first principal component of each module summarizing the gene expression patterns in each module. Once the modules were assigned, gene expression relatedness was calculated by their correlation and visualized in the hierarchical clustering dendrogram in Figure 1. Each branch of the dendrogram represents an ME. Modules that are closer on the tree have more similar eigengene co-expression patterns. The height at which the branches merge indicates the level of similarity. Modules with high similarity have lower heights (shorter branches), are grouped together, and may share similar biological functions or be co-regulated.

Conversely, modules at larger heights are less similar, have more distinct expression profiles, and may be functionally different. The initial module assignment contained several modules. We merged modules based on a 0.25 cut-off threshold, resulting in module genes that are at least 75% similar. This reduces redundancies and ensures the most significant modules are assessed. In addition, larger modules are more stable and reproducible across datasets.

The modules were numbered from the largest to smallest number of genes M1-M25, as shown in Table 1. The dendrogram shows that the Green module is closely related to the Dark-Green and White modules.

**Figure 2:**
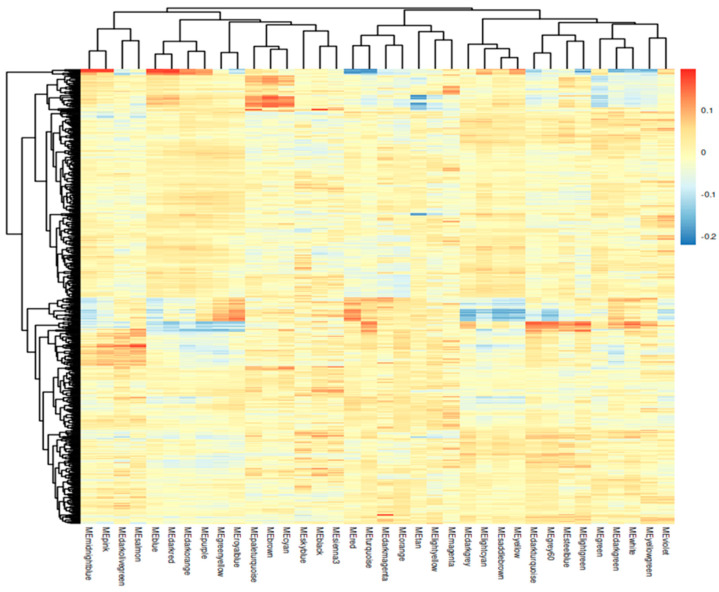
Adjacency heatmap. Each row and column of the heatmap represents a gene in the network. The genes are ordered to group highly co-expressed genes according to their hierarchical clustering. The color of each cell in the heatmap corresponds to the adjacency value between a pair of genes. Blue indicates low adjacency or weak co-expression. Red has high adjacency and strong co-expression.

In the adjacency matrix, we visualized the pairwise relationships between genes within a network. This displays the strength of the co-expression relationships between the genes and allows for an inspection of the gene relationships and identification of clusters of highly expressed genes. The heatmap in Figure 2 is a graphical representation of the adjacency matrix. The adjacencies are computed using the Pearson correlation method and transformed into adjacency values using the soft-threshold power. This method emphasizes strong correlations and reduces weak correlations. Overall, the adjacency matrix aids in the identification of biologically meaningful patterns and functional gene groups.

**Figure 3:**
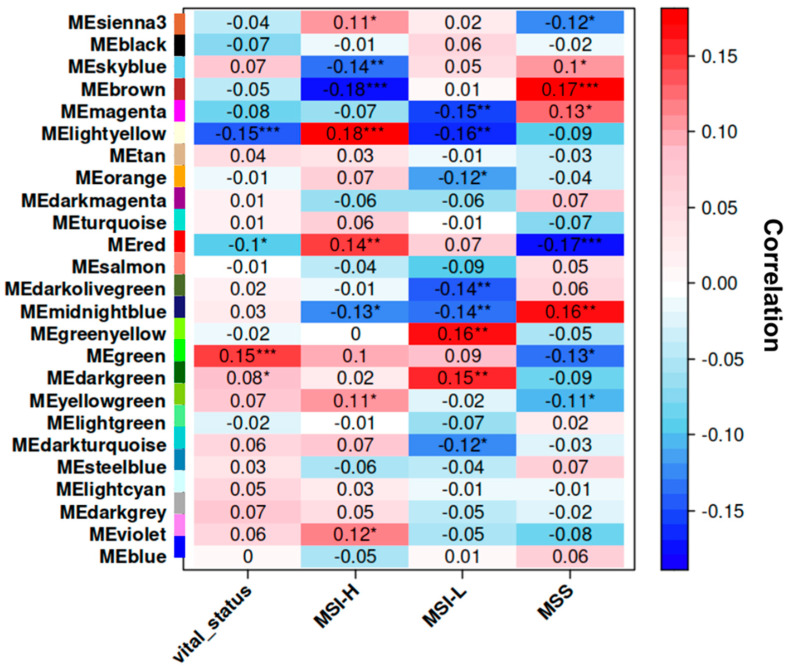
WGCNA Module–trait relationship map. This heatmap shows the relationship between clinical traits and unique modules. The color intensity represents the strength of the correlation, with red indicating a positive correlation and blue a negative correlation. Statistical significance levels are indicated by * *p* < 0.05, ** *p* < 0.01, ***, and *p* < 0.001. In vital_status, MEgreen (M5) has an R = 0.15. This indicates a moderate positive correlation with vital status and strong association with survival outcomes. MEdarkgreen also displays a statistically significant positive association (R = 0.08). MElightyellow (R = − 0.15) and MEred (R = − 0.1) have a statistically significant negative correlation to vital status. Additionally, the matrix includes microsatellite instability (MS) high, low, and stable (H, L, S), providing a comprehensive overview of how these gene modules interact with tumor characteristics and patient outcomes.

Module–trait relationship correlation was assessed using the Pearson coefficient (R) and student *t*-test *p*-values to quantify the strength and significance of associations between MEs and clinical traits. Figure 3 depicts the statistical significance of the correlations. Among the identified modules, MEgreen and MEdarkgreen exhibited a moderate positive correlation with vital status (r = 0.15 and r = 0.08), suggesting that the genes within these modules are associated with patient survival. Conversely, the genes in the MElightyellow and MEred are negatively correlated with the patient’s vital status (r = − 0.15 and − 0.1). For subsequent analysis, we will focus on the most significant modules, Green and Light Yellow.

**Figure 4:**
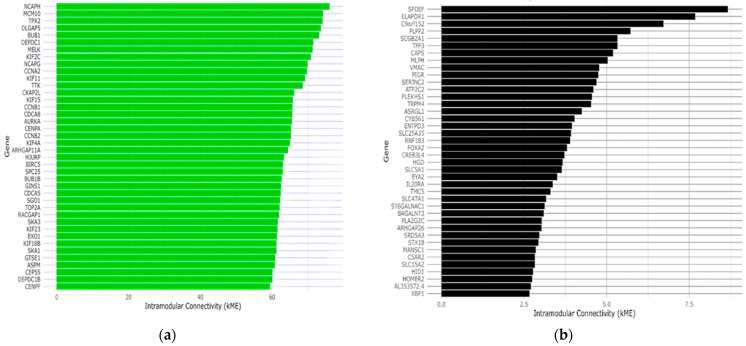
Endometrial cancer patient vital status hub genes identified by WGCNA. Visualization showing the top 40 positively or negatively correlated genes in significant modules ranked by their intramodular connectivity (*kME*). (**a**) The genes highlighted by the green color depict the top positively correlated genes in the Green module associated with vital status. These genes are involved in cell cycle regulation and mitosis, which include *NCAPH*, *MCM10*, *TPX2*, and *DLGAP5*; (**b**) the Light-Yellow module, depicted by the black bars, is negatively associated with patient vital status. The genes in this module include *SPDEF*, *ELAPOR1*, and *C9orf152*. They are involved in mucosal immunity and epithelial cell differentiation.

Intramodular connectivity, also known as module membership, was assessed using *kME* (*k* within module eigengene) values. This measure is used to quantify how connected or central a gene is within a module and is represented by this equation.

$${kME}_{i}=cor(x_{i},\;{ME}_{M})$$

where $x_{i}$ represents the gene expression of gene *I* and *ME* is the module eigengene of module *M*. This method is crucial for identifying hub genes that may play a significant role in the module’s network. Our intramodular connectivity assessment identified the top 40 genes (Figure 4) in the Green and Light-Yellow modules that may be key drivers of patient vital status. Subsequent analysis of these hub genes allowed for a more in-depth investigation of biological pathways and networks. This selection increased the statistical power of downstream analyses, increasing the likelihood of identifying significant biological trends, and permitted the evaluation of both high-impact and moderately impactful genes, providing a more robust and reliable analysis of the molecular mechanisms driving EC. In addition, the genes with a high *kME* may have similar functions or are involved in the same biological pathways. The Green module was found to be positively correlated with patient vital status. The hub genes for the Green module include *NCAPH*, *MCM10*, *TPX2*, *DLGAP5*, *BUB1*, *DEPD1*, *MELK*, *KIF2C*, *NCAPG*, *CCNA2*, *KIF11*, *TTK*, *CKAP2L*, *KIF15*, *CCNB1*, *CDCA8*, *AURKA*, *CENPA*, *CCNB2*, and *KIF4A*. These genes are essential for the progression through the cell cycle, mitotic spindle formation, and chromosome segregation. The overexpression of these genes may contribute to uncontrolled proliferation and poor prognosis. Additionally, the genes in the Light-Yellow module include *SPDEF*, *ELAPOR1*, *C9orf152*, *PLPP2*, and *SCGB2A1* and may be involved in several biological processes including tumor immunity and epithelial cell differentiation.

### 2.2. STRING

WGCNA identified a set of hub genes from the modules that were significantly correlated with patient vital status. Subsequently, Search Tool for the Retrieval of Interacting Genes/proteins (STRING) analysis was conducted to investigate the protein–protein interaction (PPI) of the top 40 hub genes from these modules with a PPI enrichment *p*-value < 1.0 × 10^−16^. Genes positively correlated with deceased EC patients (green nodes) and genes negatively associated with patient vital status (yellow nodes) were mapped to visualize and assess possible functional relationships and regulatory pathways.

**Figure 5:**
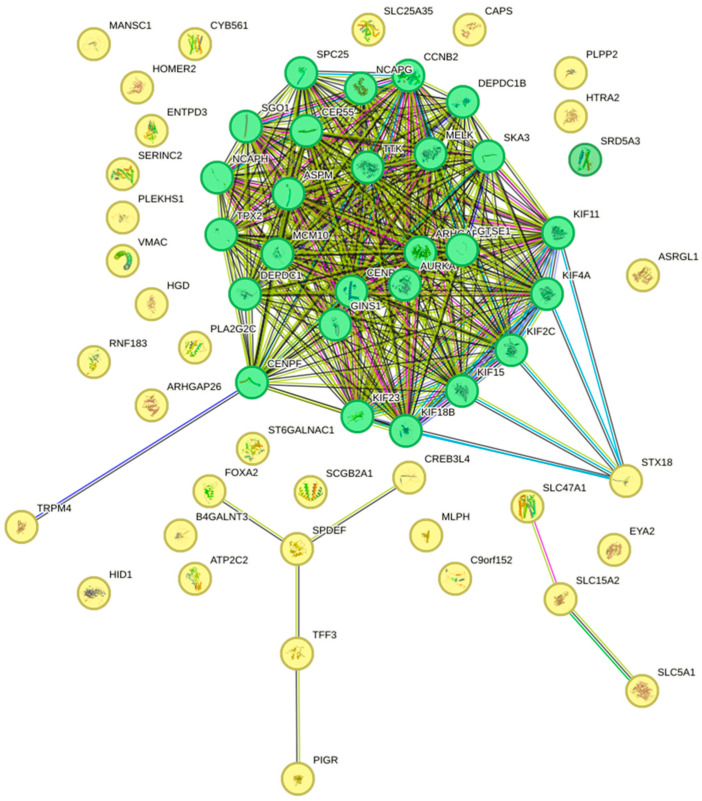
STRING network analysis of hub genes. The Green module hub genes are represented by the green dots and are highly interconnected. This suggests that they may be involved in common pathways. Conversely, the Yellow hub genes are not as interconnected as compared to the Green module genes. Thicker edges represent stronger interactions, whereas thinner edges are weak interactions. This may indicate that these genes are involved in a variety of functions such as the immune response and tumor suppression.

This network revealed distinct clustering of the Green hub genes (Figure 5). These genes formed a highly interconnected network, with key central nodes such as AURKB, *CEP55*, *CCNB2*, and *MCM10*, suggesting their involvement in common pathways potentially driving aggressive tumor behavior and contributing to poor patient prognosis.

In contrast, the Yellow hub genes depicted less interconnectivity and were associated with various biological processes. *FOXA2*, *PIGR*, and *SPDEF* were shown to be key nodes involved in the immune response and cellular differentiation pathways These results suggest that immune surveillance and cellular differentiation may contribute to improved survival outcomes [12,13,14].

*TRPM4* and *STX18* are Light-Yellow hub genes connected to the Green hub genes, suggesting a functional crosstalk between cell cycle regulation and processes like immune modulation, vesicular trafficking, and cell differentiation. These genes may mediate the integration of signals from the tumor growth mechanism (Green module) and modulate the environmental response. This response may include immune cell activity or epithelial differentiation (Yellow module). This crosstalk could be crucial for tumor survival, immune evasion, or tumor immunity.

### 2.3. Differential Gene Expression

**Figure 6:**
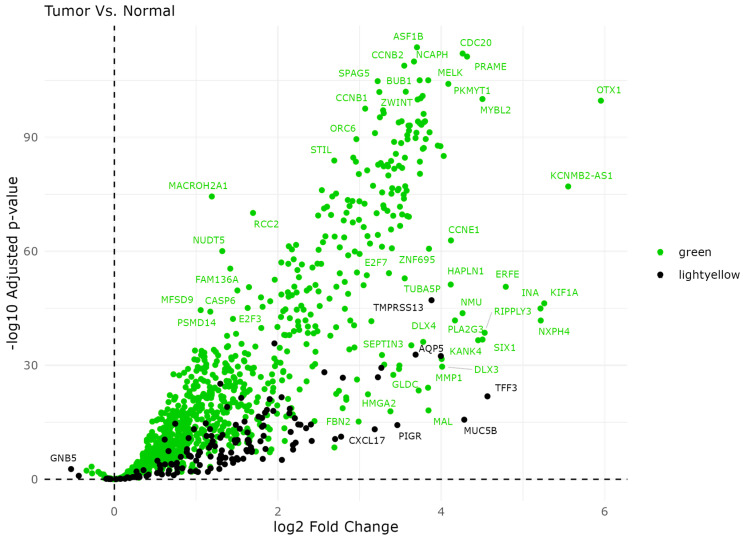
Volcano plot of module gene expression identified in WGCNA. This figure illustrates the differential expression in tumor vs. normal tissue for all genes in the Green and Light-Yellow modules. The green points reflect the genes in the Green module, whereas the black points represent the Light-Yellow module genes. The labeled genes represent the most significant log2 fold change and the − log adjusted *p*-value.

We performed differential gene expression analysis to identify differentially expressed genes between tumor and normal tissue samples, as shown by 957 genes in the Green module and 146 genes in the Light-Yellow module (Figure 6). The DEGs were further sorted based on statistical significance, considering both the *p*-value and log fold change. This allowed us to focus on genes that exhibit robust and biologically meaningful differences between the two sample groups. We hypothesize that the top hub genes identified in WGCNA will also rank among the most significantly differentially expressed genes.

In the Green module, several genes exhibit significant upregulation. ASF1B has the highest log2 fold change and significant *p*-value (<0.01). Other genes such as BUB1, *NCAPH*, *CDC20*, *KIF1A*, and *CCNB1* also show significant upregulation, suggesting their potential involvement in critical pathways that may contribute to disease progression and poor patient survival outcomes. The negative correlation to the WGCNA patient vital status in the Light-Yellow module suggests that a higher expression of these genes is associated with better patient outcomes, potentially indicating protective roles or involvement in pathways that improve survival rates. Genes such as *TMPRSS13*, *AQP5*, *TFF3*, *PIGR*, and *MUC5B* were differentially expressed in the Light-Yellow module.

Our differential gene expression analysis provides insights into the molecular alterations underlying endometrial tumorigenesis, highlighting potential biomarkers and therapeutic targets for further investigation.

### 2.4. Gene Ontology

Gene ontology (GO) analysis provides insights into the functional significance of the differentially expressed genes, highlighting key biological processes, cellular components, molecular functions, and pathways dysregulated in a given disease. To gain insight into the biological processes underlying the differentially expressed genes (DEGs), we conducted a GO analysis, which revealed several key biological processes. The top biological processes among DEGs for the Green module were chromosome segregation and mitotic nuclear division. This suggests that the dysregulation of DNA replication processes may play a crucial role in endometrial cancer tumorigenesis, potentially influencing cellular proliferation, differentiation, and migration within the tumor microenvironment. Lastly, the Light-Yellow module is involved in glycosylation processes. These processes are crucial for cell division and proliferation, and their enrichment among the DEGs highlights their potential roles in driving EC progression.

### 2.5. Survival Analysis

Survival analysis was conducted to evaluate the impact of DEGs on patient survival outcomes. The Kaplan–Meier survival analysis model was employed using Survminer (V0.49) and Survival (V3.57) in R to assess the association between gene expression levels and overall survival times, considering vital status (Dead) as the event of interest. The differentially expressed hub genes positively correlated with patient vital status from the Green and Light-Yellow modules (*p* < 0.05 *kME* > 5.0, log fold change (LFC) ≥ 1.5). Patients were stratified into high- and low-expressing groups based upon the median values and compared for statistical significance (*p* < 0.05).

**Figure 7:**
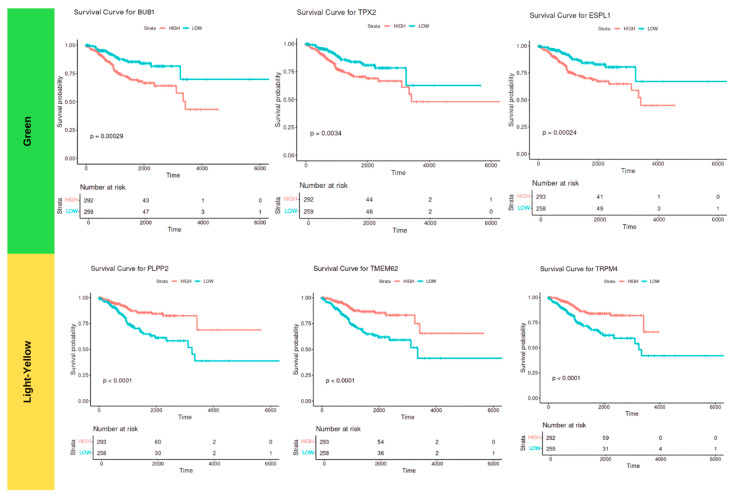
Kaplan–Meier plots and ROC curves. The top 40 WGCNA hub genes underwent survival analysis using the Kaplan–Meier analysis and were evaluated using ROC-AUC. Through this analysis, we were able to identify the top 3 genes that possessed moderate predictive value (*p* < 0.05). The Green module indicated that *TPX2*, *BUB1*, and *ESPL1* could serve as possible prognostic genes for both tumor development and poor patient outcomes. The Light-Yellow module suggests that *TMEM62*, *PLPP2*, and *TRPM4* could be involved in protective mechanisms and better patient outcomes.

Among the top upregulated DEGs, *TPX2*, *ESPL1*, and *BUB1* in the Green module and *PLPP2*, *TMGM62*, and *TRPM4* in the Light-Yellow module exhibited a statistically significant impact on patient survival (Figure 7). Of these genes, *PLPP2* also had the greatest prognostic value due to its presence in the top DEGs and hub genes.

These findings in Table 2 highlight the prognostic significance of specific DEGs in endometrial cancer, providing insight into potential biomarkers for patient risk stratification and therapeutic targeting. Six key genes were identified from two significant WGCNA modules.

Within the Green module, *TPX2*, *ESPL1*, and *BUB1* demonstrate associations with patient outcomes, with *p*-values indicating statistical significance and AUC values ranging from 0.62 to 0.65. These results suggest that these genes may contribute to patient survival classification. Similarly, the Light-Yellow module genes *TRPM4*, *TMEM62*, and *PLPP2* show moderate AUC values supporting their possible role in predicting patient survival outcomes. Further validation studies are needed to confirm the clinical utility of these genes in predicting patient outcomes and guiding personalized treatment strategies.

**Figure 8:**
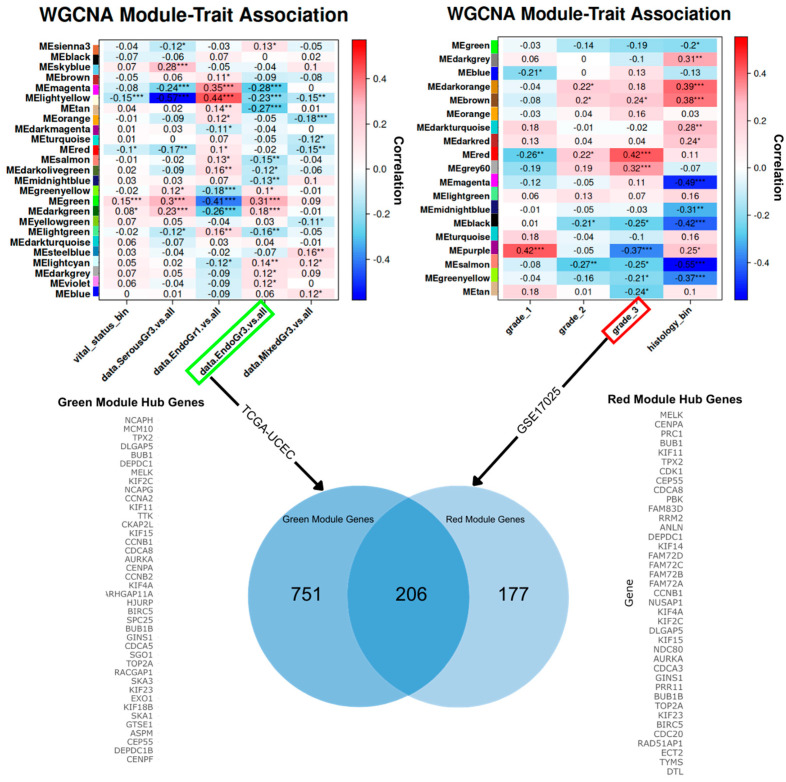
Overlap WGCNA module-trait relationship map of Green module in TCGA dataset and Red module in the GEO dataset. WGCNA analysis indicates Grade 3 tumors are most significantly correlated with the Green module (vital status) in the TCGA-UCEC dataset. Statistical significance levels are indicated by * *p* < 0.05, ** *p* < 0.01, ***, and *p* < 0.001. Using the GSE17025 data, we aimed to validate our Green module genes. We found that the Grade 3 tumors in this dataset corresponded with the Red module. After matching genes between datasets, N/A values were removed, leaving 751 Green module genes (TCGA-UCEC) and 177 (GSE17025) genes. Both WGCNA groups had 206 genes in common. This supports our findings that these genes are also implicated in tumor progression and patient survival.

Our validation analysis identified 206 overlapping hub genes between the Red (GEO) and Green (TCGA-UCEC) modules that were previously associated with Grade 3 tumors and patient vital status. This significant overlap suggests that there is a potential link between high-grade tumors and survival outcomes in UCEC. As shown in Figure 8, the TCGA-WGCNA correlation matrix highlights a significant correlation between the Green module and Grade 3 tumors. Moreover, the GEO module–trait correlation matrix indicated that the Red module was the most significantly positively correlated with Grade 3 tumors. The Venn diagram visualizes the overlap. This suggests that shared genes are potentially relevant to both tumor progression and prognosis.

**Figure 9:**
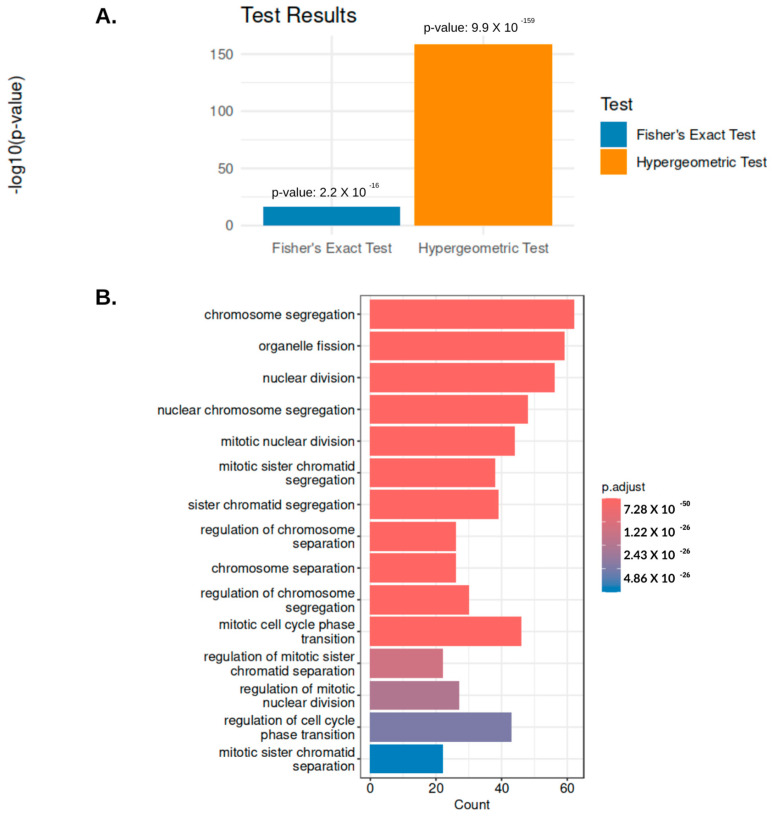
Visualization of the statistical and functional validation of module genes. (**A**) The Red and Green module genes were subject to the hypergeometric test (*p* = 9.9 × 10^−159^) and Fisher’s exact test *p* = 2.2 × 10^−16^. These results emphasize the statistical significance of the overlapped genes and the biological function of these genes in endometrial cancer. (**B**). Depicts the enriched GO terms such as chromosome segregation, organelle fission, and nuclear division. These processes may play a critical role in EC.

To statistically validate the overlap, we conducted a hypergeometric test (*p* = 9.90 × 10^−159^). These results indicate the overlap was highly unlikely to have occurred by chance. We supported this analysis with the Fisher’s exact test, with *p* = 2.2 × 10^−16^ and an odds ratio greater than 23. This indicates that there is a strong association between the genes in these modules. Together, these findings suggest that the overlapping genes are functionally relevant to UCEC. Additionally, GO analysis was conducted to investigate biological processes associated with the common genes. Chromosome segregation, organelle fission, and nuclear division were some of the enriched terms, as shown in Figure 9.

The combined statistical validation and GO enrichment analysis illustrates the statistical strength of these overlapping genes and biological relevance in UCEC.

## 3. Discussion

The comprehensive analysis of gene expression patterns in endometrial cancer provides insights into the molecular mechanisms underlying tumorigenesis and patient outcomes. In this study, we used a multi-faceted approach, integrating differential gene expression analysis, gene ontology enrichment, and survival analysis to elucidate the biological significance of dysregulated genes and their potential implications for disease prognosis and treatment. We highlight the central role of cell cycle regulation, mitotic spindle assembly, and intercellular signaling in endometrial cancer progression. Our findings corroborate previous studies that have implicated *TPX2*, *BUB1*, and *ESPL1* in the development and progression of endometrial cancer [15,16,17,18,19,20,21,22]. Our analysis supports that these genes are associated with poor outcomes in EC, consistent with previous multi-omics studies [23]. Our study builds upon the applications of WGCNA in endometrial cancer where Chou et al. and Zhu et al. demonstrated the utility of gene co-expression network analysis in endometrial cancer [24,25]. While these studies investigated cancer-related modules, our work provides deeper insights into the molecular mechanisms by integrating STRING network analysis. This additional layer of network analysis further supports direct or indirect interactions and adds functional relevance to the co-expressed genes. Unlike studies that identified specific prognostic markers (e.g., *ANO1*, *TICRR*, and *SKA3*), our study highlights clinically significant gene modules that correlate with survival and clinical traits [26]. This approach emphasizes enriched biological processes that provide a new perspective on potential therapeutic targets.

Weighted gene co-expression analysis is a useful tool in identifying co-expressed and functionally related genes associated with clinical traits. Previous studies have used WGCNA to investigate gene co-expression in association with phenotypic characteristics of EC such as type, stage, and grade [22,25]. The 2014 study revealed that *TP53*, *BUB1*, *AURKB*, and *CENPA* among other hub genes were potentially prognostic genes in EC [24]. Additional co-expression analysis studies have shown that *AKT1*, *TICRR*, *PPIF*, *ANO1*, and *PTGDS* are possible regulators of endometrial cancer tumorigenesis [27,28,29]. Our WGCNA analysis identified 957 genes in the Green module significantly positively associated with patient vital status. These genes are most associated with regulatory networks impacting patient vitality. Of those genes, the top 40 hub genes were assessed. These genes are primarily involved in cell cycle regulation and mitosis. Specifically, cyclin A2 (CCNA2) binds to CDK2 and cyclin-dependent kinase 1 (CDK1), forming a regulatory complex for the S phase and the G2/M transition of the cell cycle [30,31]. The overexpression of *CCNA2* can lead to uncontrolled cell division [32]. Cyclin B1 (CCNB1) also forms a complex with *CDK1*, initiating mitosis [32]. Overexpression can lead to the premature entry of mitosis, leading to genomic instability, as shown in the promotion of breast cancer [33]. Similarly, Minichomosome Maintenance 10 (*MCM10*) is required for the initiation of DNA replication before mitosis [34,35,36]. *MCM10* may also work to load and stabilize the replication fork [36,37]. During mitosis, targeting protein for Xklp2 (*TPX2*) is critical for spindle assembly and the organization of microtubules into a bipolar spindle [38,39]. For example, it binds and activates *AURKA* and localizes it to the spindle [38,39,40]. Kinesin family genes *KIF11* and *KIF15* work together for spindle formation and chromosome alignment [41]. It traverses down microtubules, separating the spindle poles [41,42]. KIF2C is a microtubule depolymerase that regulates microtubules and is required for spindle function and chromosome segregation [42,43,44].

In non-cancerous cells, there is a functional spindle assembly checkpoint. Budding Uninhibited by Benzimidaloes 1 (*BUB1*) is a crucial component of the spindle assembly checkpoint, ensuring that chromosomes are properly attached to the spindle microtubules before the cell proceeds to anaphase [45,46]. Similarly, Threonine Tyrosine Kinase (*TTK*) plays a key role in the spindle checkpoint by preventing aneuploidy by assessing chromosomal attachment to the spindle [47,48,49]. Non-SMC Condensin 1 Complex subunits H and G (*NCAPH* and *NCAPG*) are part of the condensing complex essential for chromosome condensation during mitosis [50,51]. In addition, Cytoskeleton-Associated Protein 2 Like (*CKAP2L*) is involved in spindle assembly and chromosome stability, ensuring chromosomes are correctly partitioned to daughter cells [51]. DEP Domain Containing 1 (DEPDC1) and Maternal Embryonic Leucine Zipper Kinase (*MELK*) are directly associated with oncogenesis and tumor progression [52,53,54,55]. *DEPDC1* is involved in cell cycle progression and promotes cancer cell survival by inhibiting apoptosis [53]. *MELK* regulates the G2/M transition of the cell cycle and promotes tumor proliferation and survival [54,55].

The WGCNA methods of using binarized clinical data provide a nuanced understanding of the gene expression profiles associated with patient outcomes. The positive correlation between the Green module with deceased patients suggests that upregulated genes within this module may be linked to adverse clinical outcomes. Specifically, the strong upregulation of cell cycle-related genes in the Green module could indicate that dysregulated cell proliferation contributes to disease progression and poorer prognosis. On the other hand, the Light-Yellow module, which is negatively correlated with deceased patients, highlights genes that might play a protective role. These findings show the importance of module-specific gene expression in predicting patient outcomes and identifying potential therapeutic targets. The positively correlated module (Green) may include genes that could serve as biomarkers for poor prognosis, while the negatively correlated Light-Yellow module points to genes that might be leveraged to improve survival rates. Table 3 highlights the key genes identified in this study.

**Figure 10:**
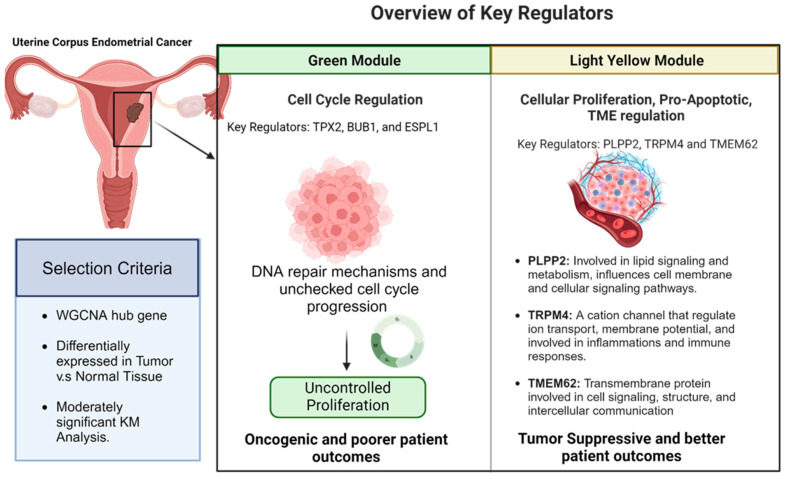
Overview of key regulators identified in the Green and Light-Yellow modules from endometrial cancer RNA-Seq data. The Green module is associated with cell cycle regulation, which may contribute to uncontrolled proliferation. The Light-Yellow module includes genes that are involved in cellular proliferation, pro-apoptotic pathways, and tumor microenvironment regulation. Key regulators in the Light-Yellow module are *PLPP2* (lipid signaling and metabolism), *TRPM4* (cation channel affecting ion transport and immune responses), and *TMEM62* (transmembrane protein involved in cell signaling). The selection criteria for the identified genes included WGCNA hub status, differential expression in tumor vs. normal tissue, and significance in Kaplan–Meier analysis.

The genes in the Green module likely contain key genes that drive tumor progression. The upregulation of *TEOC2*, *CDC20*, and *CCNB1* in the Green module suggests that these genes may play roles in promoting unchecked cells. The genes in the Green module likely contain key genes that drive tumor progression, The upregulation of *TEOC2*, *CDC20*, and *CCNB1* in the Green module suggests that these genes may play roles in promoting unchecked cell proliferation, genomic instability, and evasion of apoptosis, all of which are hallmarks of cancer [56,57]. For instance, *CDC20*, a known regulator of the cell cycle, may facilitate rapid tumor cell division, while *CCNB1*’s involvement in the G2/M transition could enhance the proliferative capacity of tumor cells. The sustained upregulation of these genes in tumor tissues, as revealed by the differential gene expression analysis, further supports their contribution to aggressive tumor behavior and poor clinical outcomes (Figure 10).

In addition to promoting tumor cell proliferation, the genes in the Green module might also influence the tumor microenvironment (TME). For example, the upregulation of immune-modulatory genes could lead to immune evasion, allowing the tumor to escape immune surveillance. This could result in a more permissive environment for tumor growth and metastasis. On the other hand, the PLPP2 identified in the Light-Yellow module may have a protective role in EC by reducing proliferation, angiogenesis, and modulating cellular immunity [58,59].

The validation analysis demonstrated a significant overlap in high-grade endometrioid tumors, and those linked to patient survival. The GEO clinical data lacked comprehensive clinical annotations compared to the TCGA dataset. Specifically, this dataset was missing patient vital status information, which is essential for conducting direct survival analysis. The literature revealed that the FIGO stage and tumor grade could be positive indicators of patient outcomes [60,61,62]. In total, 206 genes were found to be present in both the TCGA-UCEC Green module and the Red module from the GEO dataset. Statistical validation through hypergeometric and Fisher’s exact test adds robustness to our findings. The observed overlap likely reflects underlying biological processes that drive EC tumor progression and patient outcomes. We contextualized the genes using GO enrichment. This analysis showed that the common genes in both datasets were involved in critical processes for cell cycle regulation and genomic stability. This is relevant because high-grade tumors or tumor progression that contributes to patient death may have disrupted chromosomal segregation and nuclear division, which could lead to increased mutation rates and genomic instability. The enrichment of these processes aligns with their potential role in promoting aggressive tumor behavior and poorer patient outcomes.

Our analysis identified two critical Light-Yellow hub genes, *TRPM4* and *STX18*, that are connected to the Green module. This intermodular connection suggests a functional crosstalk between cellular proliferation and processes related to immune modulation and vesicular transport. *TRPM4* is known for its role in ion transport and immune response regulation [63]. It may act as a mediator between the rapidly proliferating cancer cells and the surrounding tumor microenvironment [63,64,65]. Its connection to the Green module genes implies that ion flux and immune modulation could play a key role in regulating tumor proliferation. *STX18* is involved in vesicular trafficking and the facilitation of protein and signal transport [66,67,68]. This function may influence tumor progression and cell– cell communication in the TME. The connection between *STX18* and the Green module genes suggests that vesicular transport may regulate the malignant phenotype by mediating communication between proliferating tumor cells and surrounding tissues. The interplay between these modules may present new therapeutic opportunities, specifically targeting immune-regulatory and vesicular trafficking pathways.

The integration of WGCNA and differential gene expression analyses provides an overview of genes that are not only differentially expressed between normal and tumor tissues but are also critically associated with patient outcomes. This dual approach allows for the identification of key molecular drivers in tumor development that directly influence survival rates. The genes identified in the Green module likely represent oncogenic drivers, while those in the Light-Yellow module may serve as tumor suppressors or protective factors.

Differential gene co-expression analysis originally revealed that *TPX2* and *BUB1* may be associated with the development of EC [69]. GO enrichment analysis revealed the biological processes and pathways associated with the DEGs. Survival analysis revealed the prognostic significance of specific genes in endometrial cancer. Genes such as *BUB1*, *TPX2*, and *ESPL1* demonstrated significant associations with patient survival outcomes and tumor progression, suggesting their potential utility as prognostic biomarkers for risk stratification and treatment selection. Yang et al. provided evidence that the overexpression of *TPX2* in functional assays promoted cancer cell progression, invasion, and migration [15]. Additionally, *TPX2* was shown to activate the CX3CR1/CXCL10 chemokine and PI3K/Akt pathways and SP1 was shown to negatively regulate *TPX2* expression [15]. Others have shown that KIF4A interacts with TPX2 to enhance its stability via the inhibition of ubiquitination for the genomic stability of EC cells during mitosis [70], which validates or confirms our findings for the important role of *TPX2*. Moreover, previous studies validate the TPX2 protein as a prognostic biomarker of EC [71]. This study illustrated the utility of bioinformatics in identifying biomarkers with significant impacts on tumor progression and prognosis. Cicirò et al. illustrated that *BUB1* was significantly overexpressed in TCGA gynecological tumors (UCS, CESC, UCEC, and OV) [18]. Their Kaplan–Meier analysis revealed that the higher expression of *BUB1* led to worse overall survival. Furthermore, they showed that an elevated BUB1 level may influence drug resistance [18]. Indeed, Kaplan–Meier curve analysis revealed *TPX2*, *BUB1*, and four other genes were significantly negatively related to the 5-year overall survival in EC patients [72]. *ESPL1* was revealed to be associated with poor prognosis in a 2024 study where overexpression promoted proliferation in an EC cell line [20]. Their results support the oncogenic role of *ESPL1* in EC.

Inhibitors for the differentially expressed genes, *BUB1*, *CDC20*, *CDC6*, *MYBL2*, *KIF14*, *SPAG5*, and *ORC6*, are currently in pre-clinical development [40,73,74,75]. The MELK inhibitor OTSSP167 has shown potential in pre-clinical models for several cancer types, including endometrial cancer [73,76]. Some potential investigational targets include the SKA1/3 kinetochore–microtubule interface, ASF1B inhibitors targeting chromatin regulation, SGO1 for mitotic regulation, the NCAPH condensing complex, and PKMYT1 cell cycle regulation. Additionally, TPX2, targeted by the CDK4/6 inhibitor Ademaciclib, has shown advancements in regulating cell cycle progression primarily in HR-positive breast cancer [77]. Alisertib targets AURKA by disrupting mitotic spindle formation and is being investigated for treating peripheral T-cell lymphoma [78,79]. Moreover, therapies targeting S1P1R, such as Fingolimod and Siponimod, are under investigation for modulating immune responses in cancer immunotherapy [80]. PLPP22/LPR2, involved in the PI3K/AKT pathway, may serve as a target for therapies like LY294002, which are being investigated for breast and prostate cancers [81,82]. The targets in Table 4 highlight avenues for intervention in cancer progression and patient survival.

## 4. Materials and Methods

### 4.1. Data Acquisition and Preprocessing

All analyses were conducted in R version 4.3 in the Jupyter environment. We obtained *n* = 553 transcriptomic Uterine Corpus Endometrial Carcinoma (UCEC) primary tumor samples and *n* = 35 matched normal endometrial tissue samples from the Cancer Genome Atlas (TCGA) using the TCGAbiolinks R package (Version 2.30.0) [83], as shown in Supplemental Figure S1. The GDCquery function was used to access the dataset using the following specified parameters: project = “TCGA-UCEC”, data. category = “Transcriptome Profiling”, and workflow. type = “STAR-counts”, sample. type = c (“Primary Tumor”, “Solid Tissue Normal”). After the data were retrieved, the GDCprepare function was employed to process and organize the data. Then, the SummarizedExperiment function was used to extract the initial gene expression matrix.

### 4.2. Preprocessing and Normalization

Subsequently, we conducted quality control on our gene expression matrix (*n* = 60,660 reads). We filtered out genes with less than 15 counts in at least 75% of samples (*n* = 441). After filtering, 14,802 genes remained. Principal components analysis (PCA) was performed to assess and visualize potential batch effects. No significant clustering was observed, indicating minimal batch effects in the dataset. We then applied variance stabilization using the VarianceStabilizingTransformation (VST) function with the blind = FALSE setting from the DESeq2 (Version 1.44.0) package [84]. VST is a necessary step because it removes technical noise and improves the compatibility of the data. This is important because it allows for an observance of differences that most likely reflect co-expression patterns and biological processes instead of sequencing artifacts. Following VST, the counts matrix was normalized using the estimateSizeFactors function to adjust for library size differences and creating a DESeqDataSet object with uniform expression levels. The normalized gene expression matrix was then transposed to align the data structure with the requirements of WGCNA analysis where the samples are the row names, and the gene ids are the columns. This transformation is required for calculating the module–trait correlations and network analysis because it allows for each gene to be its own variable in relation to the sample traits.

### 4.3. Gene Clustering and Network Analysis

#### Clinical Trait Binarization

The clinical data were accessed through the TCGABiolinks gdc_query function. The clinical data were matched with the expression data based on the submitter_id. Clinical traits, such as vital status and MSI marker status, were binarized for statistical association in the WGCNA. For example, Vital status “1” = “Dead” and “0” = “Alive”, as shown in Table 5.

Weighted gene co-expression network analysis (WGCNA) is a robust bioinformatics tool used to identify and interpret gene expression patterns in high-dimension genomic data [85]. It calculates the eigengene, Pearson coefficient, and *p*-values for each module and correlates the first principal component of each module to the clinical traits. WGCNA (Version 1.72.5) was installed from the Comprehensive R Archive Network (CRAN). Using the pickSoftThreshold function, we tested powers from 1 to 50 to determine a power with the ability to achieve a scale-free topology fit index above 0.8. Based on our data, we selected the power of 9 to meet this threshold. This approach aligns with established practices in WGCNA, as demonstrated in previous studies [86,87]. By using this threshold, it ensures our results are consistent with the methodological rigor in previous WGCNA studies and aids the biological interpretability of our subsequent analysis.

The network was constructed using the WGCNA blockwiseModules function with the following parameters: WGCNA dynamic tree-cutting algorithm, cuttreeDynamic, power = 9, dendro = geneTree, dist-M = dissTOM, deepsplit = 2, minModuleSize = 30, mergeCutHeight = 0.25, corType = “*p*”, networkType = “signed”, and verbose = “5”.

### 4.4. STRING Network Analysis

WGCNA hub gene interactions were analyzed using STRING Version 11.5, https://string-db.org/ (accessed on 20 September 2024). The list of hub genes was uploaded to STRING, with the species set to Homo sapiens. The interaction score was set to a minimum confidence threshold of 0.7 (high confidence). K-means clustering was applied to identify clusters of interacting genes (PPI enrichment *p*-value < 1.0 × 10^−16^).

### 4.5. Differential Gene Expression Analysis

Differential gene expression (DEG) analysis between endometrial cancer samples (*n* = 553) and normal endometrial tissue samples (*n* = 35) was performed using DESeq2 with 14,802 features [88]. DESeq2 (Version 1.42.1) is an R package used for analyzing count data from high-throughput sequencing assays, such as RNA-Seq. DESeq2 was employed to identify genes that were significantly upregulated or downregulated in endometrial cancer compared to normal tissue, with a predefined threshold of an adjusted *p*-value < 0.01 and |log2 fold change| > 1.5. We set our statistical significance threshold to 0.01 and applied the Benjamin–Hochberg correction to account for multiple comparisons. This approach ensures that the differentially expressed genes are rigorously assessed while maintaining discovery and statistical reliability. The gene modules significantly correlated with patient survival from WGCNA were used to filter the DEG analysis and visualized with the EnhancedVolcano (Version 1.20.0) R package.

### 4.6. Gene Ontology Analysis

The differentially expressed genes identified from the previous step were subjected to gene ontology analysis to elucidate the biological processes associated with endometrial cancer progression. This analysis was performed using the ClusterProfiler R (Version 4.10.1) package, with a significant threshold of an adjusted *p*-value < 0.05.

### 4.7. Survival Analysis

Survival analysis was conducted using Survminer (Version 0.49) and Survival (Version 3.57) to assess the association between the expression levels of DEGs and patient survival outcomes. Patients were stratified into high-expression and low-expression groups based on the median expression levels of the individual genes, and Kaplan–Meier survival curves were generated. The Log-Rank test was used to evaluate the significance of differences in survival between the two groups, with a *p*-value < 0.05 considered statistically significant.

### 4.8. ROC Analysis

The top differentially expressed genes positively and negatively correlated with EC patient vital status (Green and Light Yellow) were analyzed using a Receiver–Operator Curve (ROC) from the pROC (Version 1.18.5) R package. *p*-values (<0.05) were calculated using 95% confidence intervals (DeLong) [89]. This analysis enables an evaluation of the predictive ability of the differentially expressed genes as an indicator of EC vital status.

### 4.9. Validation Analysis

We conducted a validation study using the Gene Expression Omnibus (GEO) GSE17025 dataset using the GEOquery R package (Version 2.70.0). We acquired expression data from 12 normal endometrial samples and 91 samples of stage 1 endometrial cancers. The gene expression data were acquired and preprocessed using the same steps as the TCGA-UCEC data. The top 14,802 most variable genes based on variance across samples were used for subsequent analysis. For the WGCNA module–trait association, we created binary columns representing each histological grade level (Grade 1, Grade 2, and Grade 3). This enabled the examination of the association of these clinical traits and gene modules. The identified module contained Probe IDs that were mapped to gene symbols using hgu133plus2.db in R. The identified validation module genes were compared with the TCGA-UCEC WGCNA vital status module to identify a cohort of overlapping genes.

To statistically validate this association between the TCGA-UCEC and GEO datasets, we performed a hypergeometric test to assess the probability of overlap given the gene set sizes. Additionally, we conducted a Fisher’s exact test to confirm the association. Lastly, we conducted gene ontology enrichment analysis using the ClusterProfiler (Version 4.10.1) R package to identify significant biological processes of the shared genes [90].

## 5. Conclusions

In our study, we utilized a comprehensive multi-omics approach to investigate the complex molecular landscape of EC. Through the analysis of TCGA-UCEC data, we identified significant gene co-expression patterns, biological processes, and pathways that contribute to our understanding of EC progression. WGCNA revealed 957 genes within the Green module that are significantly associated with patient vital status. Our findings highlight the central role of critical cellular functions such as cell cycle regulation, mitotic spindle assembly, and intercellular signaling. The differential expression analysis further highlighted several key genes including TPX2, BUB1, and ESPL1 as key drivers of EC progression. These genes are implicated in essential oncogenic processes such as chromosomal segregation, DNA replication, and mitotic regulation, highlighting their potential role in tumorigenesis and poor patient prognosis. Gene ontology enrichment analysis revealed crucial biological processes and pathways associated with EC, such as chromosome segregation, mitotic nuclear division, and microtubule motor activity. Our survival analysis identified several differentially expressed genes with significant associations with patient survival outcomes, suggesting their utility as prognostic markers for risk stratification and treatment selection. Although not all of the genes in the WGCNA vital_status cohort (Green *n* = 957 and Light Yellow *n* = 146 modules) have prognostic value in this study, some may be possible predictive markers for targeted combination or adjuvant therapies. For example, the intermodular crosstalk between Light-Yellow genes, TRPM4 and STX18, point to interactions between tumor proliferation and immune responses/vesicular trafficking. While TRPM4 was found to be significant in our survival analysis, both of these genes may be important for modulating the TME through immune regulation and protein transport. Targeting these intermodular connections may offer a novel therapeutic strategy.

### 5.1. Limitations

The strengths in our study lie in its large sample size and the comprehensive representation of EC tumor phenotypes, providing a robust framework for identifying key molecular drivers of EC. However, we had several limitations. First, our analysis utilizes TCGA-UCEC data, which have missing clinical annotations. The independent validation using GEO (GSE17025) also had missing clinical annotations such as patient survival data, which are necessary for direct survival analysis. In addition, this study focuses on the UCEC subtype, and biomarkers may not be the same for rare and underrepresented subtypes. Furthermore, our analysis identified prognostic markers using bioinformatic methods. Functional validation experiments are needed to confirm the biological relevance and clinical utility in EC. Future studies should be aimed at addressing these limitations and build on our findings to advance precision medicine in endometrial cancer.

### 5.2. Future Directions

This study provides a computational approach to identify prognostic biomarkers. Future research directions should focus on validating these biomarkers in independent cohorts and exploring their functional roles in EC pathophysiology. For instance, in vitro cell line studies or patient-derived xenografts could be used to validate the biological relevance of these biomarkers in endometrial cancer. Since this study mainly focuses on transcriptomic data, multi-omic approaches like those mentioned by Boroń et al. could be used in future studies to gain a more in-depth picture of the biological processes contributing to tumor progression [23]. As discussed in the introduction, there are cancer health disparities among endometrial cancer patients. Like many cancer datasets, the TCGA-UCEC data had a limited racial diversity patient distribution, as shown in Supplemental Table S1. Future studies that investigate differences in gene expression between racial groups may provide insight into gene expression patterns that contribute to the health disparities. Additionally, investigating potential therapeutic targets among the identified differentially expressed genes could pave the way for novel treatment strategies, ultimately improving patient outcomes and advancing precision oncology in endometrial cancer. 

## Figures and Tables

**Table 1:** Modules in WGCNA analysis.

Order	Module Colors	# of Genes
M1	Turquoise	2888
M2	Blue	2727
M3	Brown	1719
M4	Light Cyan	1277
M5	Green	957
M6	Midnight Blue	850
M7	Red	753
M8	Black	687
M9	Magenta	658
M10	Green Yellow	518
M11	Tan	273
M12	Dark Turquoise	256
M13	Salmon	249
M14	Dark Green	200
M15	Light Green	148
M16	Light Yellow	146
M17	Dark Gray	99
M18	Orange	93
M19	Sky Blue	64
M20	Steel Blue	47
M21	Violet	46
M22	Dark Magenta	39
M23	Dark Olive Oreen	39
M24	Sienna3	35
M25	Yellow Green	34

**Table 2:** ROC AUC scores for the top 3 module genes based on their intermodular connectivity.

Module Name	Gene	*p* Value	AUC
Green	*TPX2*	0.0039647	0.6493689
	*ESPL1*	0.0003482	0.6262506
	*BUB1*	0.0004155	0.6235858
Light Yellow	*TRPM4*	2.68 × 10^−5^	0.6462833
	*TMEM62*	3.74 × 10^−6^	0.6381019
	*PLPP2*	1.52 × 10^−5^	0.6324451

The input genes were of the top 40 hub genes and top 40 differentially expressed genes among 957 Green module genes and 146 Light-Yellow module genes.

**Table 3:** Overview of potential prognostic hub genes.

Module	Gene	Hub Gene	Significant AUC Score	Log FC > 1.5
Green	*TPX2*	✓	✓	✓
	*ESPL1*	✓	✓	✓
	*BUB1*	✓	✓	✓
Yellow	*TRPM4*	✓	✓	✓
	*TMEM62*	✓	✓	✓
	*PLPP2*	✓	✓	✓

**Table 4:** Clinical and regulatory matrix of targeted therapies.

Target	Therapy	Types of Cancer Treated	Mechanism of Action	FDA Approval Status	Drug Class/Category	Combination Therapies
TPX2	Ademaciclib	Breast cancer (HR-positive, HER2-negative advanced, or metastatic breast cancer)	Inhibits CDK4/6, preventing cell cycle progression from G1 to S phase	FDA-approved	CDK4/6 inhibitor	Often combined with hormone therapy like Fulvestrant
AURKA	Alisertib	Relapsed or refractory peripheral T-cell lymphoma (PTCL), neuroblastoma, and other cancers	Inhibits Aurora kinase A, disrupting mitotic spindle formation, leading to apoptosis in cancer cells	Not FDA-approved	Aurora kinase inhibitor	Studied with chemotherapy agents like paclitaxel
S1P1R	Fingolimod, Siponimod, Ozanimod, Ponesimod	Multiple sclerosis (Fingolimod and Siponimod), studied in cancer immunotherapy for solid tumors	Modulates S1P receptor, preventing lymphocyte egress from lymph nodes, reducing immune activity	FDA-approved Under investigation for cancer	S1P receptor modulators	Investigated with immune checkpoint inhibitors (e.g., anti-PD1 therapies)
PLPP2/LPR2	Orthovanadate, Okadaic Acid, LY294002	Experimental studies in breast, prostate, and ovarian cancers (due to involvement in PI3K/AKT pathway)	PI3K/AKT pathway inhibitors, block protein phosphatase activity, leading to apoptosis in cancer cells	Not FDA-approved	Phosphatase inhibitors/PI3K inhibitors	Studied in combination with other targeted therapies like PI3K inhibitors

**Table 5:** Overview of clinical traits in analysis.

Clinical Traits	Classification
Vital Status	“Alive”, “Dead”
MSI Marker Status	MSI-H, MSS, MSI-L, Indeterminant

## Data Availability

The Uterine Corpus Endometrial Carcinoma (UCEC) data analyzed in this study were obtained from The Cancer Genome Atlas (TCGA) through the TCGABiolinks R Package (Version 2.30.0). The TCGA UCEC dataset is publicly available from the Genomic Data Commons (GDC) Data Portal at https://portal.gdc.cancer.gov/ (accessed on 27 August 2024), in accordance with TCGA’s data usage policies.

## Supplementary Materials

The following supporting information can be downloaded at: https://www.mdpi.com/article/10.3390/ijms252212356/s1.

## References

[1] Siegel R.L., Miller K.D., Wagle N.S., Jemal A. (2023). Cancer statistics, 2023. CA Cancer J. Clin..

[2] National Cancer Institute (2018). Surveillance, Epidemiology, and End Results (SEER) Program.

[3] Sung H., Siegel R.L., Rosenberg P.S., Jemal A. (2019). Emerging cancer trends among young adults in the USA: Analysis of a population-based cancer registry. Lancet Public Health.

[4] Lang S., Liao C.-I., Somasegar S., Johnson C., Darcy K., Tian C., Kapp D., Chan J.K. (2023). Trends in Uterine Cancer Mortality in the United States: A 50-Year Population-Based Analysis. Obstet Gynecol..

[5] Amant F., Moerman P., Neven P., Timmerman D., Van Limbergen E., Vergote I. (2005). Endometrial cancer. Lancet.

[6] Kandoth C., Schultz N., Cherniack A.D., Akbani R., Liu Y., Shen H., Robertson A.G., Pashtan I., Shen R., Cancer Genome Atlas Research Network (2013). Integrated genomic characterization of endometrial carcinoma. Nature.

[7] Boyiadzis M.M., Kirkwood J.M., Marshall J.L., Pritchard C.C., Azad N.S., Gulley J.L. (2018). Significance and implications of FDA approval of pembrolizumab for biomarker-defined disease. J. Immunother. Cancer.

[8] O’Malley D.M., Bariani G.M., Cassier P.A., Marabelle A., Hansen A.R., Acosta A.D.J., Miller W.H., Safra T., Italiano A., Mileshkin L. (2022). Pembrolizumab in Patients with Microsatellite Instability—High Advanced Endometrial Cancer: Results From the KEYNOTE-158 Study. J. Clin. Oncol..

[9] Mirza M.R., Chase D.M., Slomovitz B.M., Christensen R.D., Novák Z., Black D., Gilbert L., Sharma S., Valabrega G., Landrum L.M. (2023). Dostarlimab for Primary Advanced or Recurrent Endometrial Cancer. N. Engl. J. Med..

[10] Eskander R.N., Sill M.W., Beffa L., Moore R.G., Hope J.M., Musa F.B., Mannel R., Shahin M.S., Cantuaria G.H., Girda E. (2023). Pembrolizumab plus Chemotherapy in Advanced Endometrial Cancer. N. Engl. J. Med..

[11] Fader A.N., Roque D.M., Siegel E., Buza N., Hui P., Abdelghany O., Chambers S.K., Secord A.A., Havrilesky L., O’Malley D.M. (2018). Randomized Phase II Trial of Carboplatin-Paclitaxel Versus Carboplatin-Paclitaxel-Trastuzumab in Uterine Serous Carcinomas That Overexpress Human Epidermal Growth Factor Receptor 2/neu. J. Clin. Oncol..

[12] Hong L., Chen M., Huang M., Chen W., Abudukeremu X., She F., Chen Y. (2024). FOXA2 suppresses gallbladder carcinoma cell migration, invasion, and epithelial-mesenchymal transition by targeting SERPINB5. Environ. Toxicol..

[13] Berntsson J., Lundgren S., Nodin B., Uhlén M., Gaber A., Jirström K. (2014). Expression and prognostic significance of the polymeric immunoglobulin receptor in epithelial ovarian cancer. J. Ovarian Res..

[14] Bao K.-C., Wang F.-F. (2022). The role of SPDEF in cancer: Promoter or suppressor. Neoplasma.

[15] Yang M., Mao X., Li L., Yang J., Xing H., Jiang C. (2024). High TPX2 expression results in poor prognosis, and Sp1 mediates the coupling of the CX3CR1/CXCL10 chemokine pathway to the PI3K/Akt pathway through targeted inhibition of TPX2 in endometrial cancer. Cancer Med..

[16] Wang J., Zheng H., He H., Meng S., Han Y., Su Z., Yan H., Zhang Y. (2022). TPX2 Serves as a Cancer Susceptibility Gene and Is Closely Associated with the Poor Prognosis of Endometrial Cancer. Genet. Res..

[17] Hao Q., Wu H., Liu E., Wang L. (2023). BUB1, BUB1B, CCNA2, and CDCA8, along with miR-524-5p, as clinically relevant biomarkers for the diagnosis and treatment of endometrial carcinoma. BMC Cancer.

[18] Cicirò Y., Ragusa D., Sala A. (2024). Expression of the checkpoint kinase BUB1 is a predictor of response to cancer therapies. Sci. Rep..

[19] Zhang H., Li Y., Lu H. (2024). Correlation of BUB1 and BUB1B with the development and prognosis of endometrial cancer. Sci. Rep..

[20] Yang Y., Sheng Y., Zheng J., Ma A., Chen S., Lin J., Yang X., Liang Y., Zhang Y., Zheng X. (2024). Upregulation of ESPL1 is associated with poor prognostic outcomes in endometrial cancer. Biomarkers.

[21] Yang Q., Yu B., Sun J. (2020). TTK, CDC25A, and ESPL1 as Prognostic Biomarkers for Endometrial Cancer. BioMed Res. Int..

[22] Huang S., Pang L., Wei C. (2021). Identification of a Four-Gene Signature with Prognostic Significance in Endometrial Cancer Using Weighted-Gene Correlation Network Analysis. Front. Genet..

[23] Boroń D., Zmarzły N., Wierzbik-Strońska M., Rosińczuk J., Mieszczański P., Grabarek B.O. (2022). Recent Multiomics Approaches in Endometrial Cancer. Int. J. Mol. Sci..

[24] Chou W.-C., Cheng A.-L., Brotto M., Chuang C.-Y. (2014). Visual gene-network analysis reveals the cancer gene co-expression in human endometrial cancer. BMC Genom..

[25] Zhu X.-L., Ai Z.-H., Wang J., Xu Y.-L., Teng Y.-C. (2012). Weighted gene co-expression network analysis in identification of endometrial cancer prognosis markers. Asian Pac. J. Cancer Prev..

[26] Ding X., Zhang Y., Qin J., Zhang J. (2024). Clinical Value Evaluation of SKA3 in Endometrial Cancer and Its Promotion of Proliferation and Migration of Endometrial Cancer Cells. Clin. Exp. Obstet. Gynecol..

[27] Huo X., Sun H., Liu Q., Ma X., Peng P., Yu M., Zhang Y., Cao D., Shen K. (2019). Clinical and Expression Significance of AKT1 by Co-expression Network Analysis in Endometrial Cancer. Front. Oncol..

[28] Yang L., Cui Y., Sun X., Wang Y. (2021). Overexpression of TICRR and PPIF confer poor prognosis in endometrial cancer identified by gene co-expression network analysis. Aging.

[29] Wang F., Wang B., Long J., Wang F., Wu P. (2019). Identification of candidate target genes for endometrial cancer, such as ANO1, using weighted gene co-expression network analysis. Exp. Ther. Med..

[30] Pagano M., Pepperkok R., Verde F., Ansorge W., Draetta G. (1992). Cyclin A is required at two points in the human cell cycle. EMBO J..

[31] Bendris N., Lemmers B., Blanchard J.-M., Arsic N. (2011). Cyclin A2 mutagenesis analysis: A new insight into CDK activation and cellular localization requirements. PLoS ONE.

[32] Bukholm I.R., Bukholm G., Nesland J.M. (2001). Over-expression of cyclin a is highly associated with early relapse and reduced survival in patients with primary breast carcinomas. Int. J. Cancer.

[33] Mughal M.J., Chan K.I., Mahadevappa R., Wong S.W., Wai K.C., Kwok H.F. (2022). Over-Activation of Minichromosome Maintenance Protein 10 Promotes Genomic Instability in Early Stages of Breast Cancer. Int. J. Biol. Sci..

[34] Langston L.D., Mayle R., Schauer G.D., Yurieva O., Zhang D., Yao N.Y., E Georgescu R., E O’Donnell M., States U. (2017). Mcm10 promotes rapid isomerization of CMG-DNA for replisome bypass of lagging strand DNA blocks. eLife.

[35] Du W., Stauffer M.E., Eichman B.F. (2012). Structural biology of replication initiation factor Mcm10. Subcell. Biochem..

[36] Merchant A.M., Kawasaki Y., Chen Y., Lei M., Tye B.K. (1997). A lesion in the DNA replication initiation factor Mcm10 induces pausing of elongation forks through chromosomal replication origins in *Saccharomyces cerevisiae*. Mol. Cell. Biol..

[37] Warren E.M., Vaithiyalingam S., Haworth J., Greer B., Bielinsky A.-K., Chazin W.J., Eichman B.F. (2008). Structural basis for DNA binding by replication initiator Mcm10. Structure.

[38] Shao T., Jiang X., Bao G., Li C., Guo C. (2022). Comprehensive Analysis of the Oncogenic Role of Targeting Protein for Xklp2 (TPX2) in Human Malignancies. Dis. Markers.

[39] Wadsworth P. (2015). TPX2. Curr. Biol..

[40] Ma H.T., Poon R.Y. (2020). Aurora kinases and DNA damage response. Mutat. Res. Mol. Mech. Mutagen..

[41] Sharp D.J., Rogers G.C., Scholey J.M. (2000). Microtubule motors in mitosis. Nature.

[42] Miki H., Okada Y., Hirokawa N. (2005). Analysis of the kinesin superfamily: Insights into structure and function. Trends Cell Biol..

[43] Hedrick D.G., Stout J.R., Walczak C.E. (2008). Effects of anti-microtubule agents on microtubule organization in cells lacking the kinesin-13 MCAK. Cell Cycle.

[44] Moore A.T., Rankin K.E., von Dassow G., Peris L., Wagenbach M., Ovechkina Y., Andrieux A., Job D., Wordeman L. (2005). MCAK associates with the tips of polymerizing microtubules. J. Cell Biol..

[45] Kim T., Gartner A. (2021). Bub1 kinase in the regulation of mitosis. Anim. Cells Syst..

[46] Bloom C.R., North B.J. (2021). Physiological relevance of post-translational regulation of the spindle assembly checkpoint protein BubR1. Cell Biosci..

[47] Miao Y., Konno Y., Wang B., Zhu L., Zhai T., Ihira K., Kobayashi N., Watari H., Jin X., Yue J. (2023). Integrated multi-omics analyses and functional validation reveal TTK as a novel EMT activator for endometrial cancer. J. Transl. Med..

[48] Thu K.L., Silvester J., Elliott M.J., Ba-Alawi W., Duncan M.H., Elia A.C., Mer A.S., Smirnov P., Safikhani Z., Haibe-Kains B. (2018). Disruption of the anaphase-promoting complex confers resistance to TTK inhibitors in triple-negative breast cancer. Proc. Natl. Acad. Sci. USA.

[49] Musacchio A. (2015). The Molecular Biology of Spindle Assembly Checkpoint Signaling Dynamics. Curr. Biol..

[50] Wang Y., Li J.-Q., Yang Z.-L., Wang L., Zhang J.-C., Sun Y.-F., Li Z.-Y., Qin L. (2022). NCAPH regulates gastric cancer progression through DNA damage response. Neoplasma.

[51] Sun Y., Wang X., Wen H., Zhu B., Yu L. (2021). Expression and Clinical Significance of the NCAPH, AGGF1, and FOXC2 Proteins in Serous Ovarian Cancer. Cancer Manag. Res..

[52] Shen L., Li H., Liu R., Zhou C., Bretches M., Gong X., Lu L., Zhang Y., Zhao K., Ning B. (2022). *DEPDC1* as a crucial factor in the progression of human osteosarcoma. Cancer Med..

[53] Harada Y., Kanehira M., Fujisawa Y., Takata R., Shuin T., Miki T., Fujioka T., Nakamura Y., Katagiri T. (2010). Cell-permeable peptide DEPDC1-ZNF224 interferes with transcriptional repression and oncogenicity in bladder cancer cells. Cancer Res..

[54] Sun X., Gao L., Chien H.-Y., Li W.-C., Zhao J. (2013). The regulation and function of the NUAK family. J. Mol. Endocrinol..

[55] Heyer B.S., Kochanowski H., Solter D. (1999). Expression of Melk, a new protein kinase, during early mouse development. Dev. Dyn..

[56] Fang L., Yu W., Zhu P., Yu G., Ye B. (2023). TEDC2 correlated with prognosis and immune microenvironment in lung adenocarcinoma. Sci. Rep..

[57] Wan L., Tan M., Yang J., Inuzuka H., Dai X., Wu T., Liu J., Shaik S., Chen G., Deng J. (2014). APC(Cdc20) suppresses apoptosis through targeting Bim for ubiquitination and destruction. Dev. Cell.

[58] Tang X., McMullen T.P.W., Brindley D.N. (2019). Increasing the low lipid phosphate phosphatase 1 activity in breast cancer cells decreases transcription by AP-1 and expressions of matrix metalloproteinases and cyclin D1/D3. Theranostics.

[59] Huang H., Cai X., Lin J., Wu Q., Zhang K., Lin Y., Liu B., Lin J. (2023). A novel five-gene metabolism-related risk signature for predicting prognosis and immune infiltration in endometrial cancer: A TCGA data mining. Comput. Biol. Med..

[60] Li H., Zhou Q., Wu Z., Lu X. (2023). Identification of novel key genes associated with uterine corpus endometrial carcinoma progression and prognosis. Ann. Transl. Med..

[61] Lakhwani P., Agarwal P., Goel A., Nayar N., Pande P., Kumar K. (2019). High-Grade Endometrial Cancer-Behaviour and Outcomes at a Tertiary Cancer Centre. Indian J. Surg. Oncol..

[62] Berek J.S., Matias-Guiu X., Creutzberg C., Fotopoulou C., Gaffney D., Kehoe S., Lindemann K., Mutch D., Concin N. (2023). FIGO staging of endometrial cancer: 2023. Int. J. Gynecol. Obstet..

[63] Borgström A., Peinelt C., Stokłosa P. (2021). TRPM4 in Cancer—A New Potential Drug Target. Biomolecules.

[64] Liu L., Lin J., He H. (2019). Identification of Potential Crucial Genes Associated with the Pathogenesis and Prognosis of Endometrial Cancer. Front. Genet..

[65] Li X.C., Cheng Y., Yang X., Zhou J.Y., Dong Y.Y., Shen B.Q., Wang J.Q., Zhao L.J., Wang Z.Q., Li X.P. (2020). Decreased expression of TRPM4 is associated with unfavorable prognosis and aggressive progression of endometrial carcinoma. Am. J. Transl. Res..

[66] Huang C.-Y., Liao K.-W., Chou C.-H., Shrestha S., Yang C.-D., Chiew M.-Y., Huang H.-T., Hong H.-C., Huang S.-H., Chang T.-H. (2020). Pilot Study to Establish a Novel Five-Gene Biomarker Panel for Predicting Lymph Node Metastasis in Patients with Early Stage Endometrial Cancer. Front. Oncol..

[67] Bassett T., Harpur B., Poon H.Y., Kuo K.-H., Lee C.H. (2008). Effective stimulation of growth in MCF-7 human breast cancer cells by inhibition of syntaxin18 by external guide sequence and ribonuclease P. Cancer Lett..

[68] Hatsuzawa K., Hirose H., Tani K., Yamamoto A., Scheller R.H., Tagaya M. (2000). Syntaxin 18, a SNAP Receptor That Functions in the Endoplasmic Reticulum, Intermediate Compartment, and cis-Golgi Vesicle Trafficking. J. Biol. Chem..

[69] Shen L., Liu M., Liu W., Cui J., Li C. (2017). Bioinformatics analysis of RNA sequencing data reveals multiple key genes in uterine corpus endometrial carcinoma. Oncol. Lett..

[70] Zhang J., An L., Zhao R., Shi R., Zhou X., Wei S., Zhang Q., Zhang T., Feng D., Yu Z. (2022). KIF4A promotes genomic stability and progression of endometrial cancer through regulation of TPX2 protein degradation. Mol. Carcinog..

[71] Coll-de La Rubia E., Martinez-Garcia E., Dittmar G., Nazarov P.V., Bebia V., Cabrera S., Gil-Moreno A., Colás E. (2021). In silico Approach for Validating and Unveiling New Applications for Prognostic Biomarkers of Endometrial Cancer. Cancers.

[72] Yuan Y., Chen Z., Cai X., He S., Li D., Zhao W. (2021). Identification of Hub Genes Correlated with Poor Prognosis for Patients with Uterine Corpus Endometrial Carcinoma by Integrated Bioinformatics Analysis and Experimental Validation. Front. Oncol..

[73] Corson T.W., Gallie B.L. (2006). *KIF14* mRNA expression is a predictor of grade and outcome in breast cancer. Int. J. Cancer.

[74] Liu X., Xu Y., Han L., Yi Y. (2018). Reassessing the Potential of Myb-targeted Anti-cancer Therapy. J. Cancer.

[75] Otto T., Sicinski P. (2017). Cell cycle proteins as promising targets in cancer therapy. Nat. Rev. Cancer.

[76] Chung S., Suzuki H., Miyamoto T., Takamatsu N., Tatsuguchi A., Ueda K., Kijima K., Nakamura Y., Matsuo Y. (2012). Development of an orally-administrative MELK-targeting inhibitor that suppresses the growth of various types of human cancer. Oncotarget.

[77] Yang L., Chen Y., Wang N., Han W. (2022). A narrative review of the clinical development of CDK4/6 inhibitor abemaciclib in breast cancer. Transl. Breast Cancer Res..

[78] Zheng D., Li J., Yan H., Zhang G., Li W., Chu E., Wei N. (2023). Emerging roles of Aurora-A kinase in cancer therapy resistance. Acta Pharm. Sin. B.

[79] Manfredi M.G., Ecsedy J.A., Chakravarty A., Silverman L., Zhang M., Hoar K.M., Stroud S.G., Chen W., Shinde V., Huck J.J. (2011). Characterization of Alisertib (MLN8237), an investigational small-molecule inhibitor of aurora A kinase using novel in vivo pharmacodynamic assays. Clin. Cancer Res..

[80] Rodriguez Y.I., Campos L.E., Castro M.G., Aladhami A., Oskeritzian C.A., Alvarez S.E. (2016). Sphingosine-1 Phosphate: A New Modulator of Immune Plasticity in the Tumor Microenvironment. Front. Oncol..

[81] Duarte A., Silveira G.G., Soave D.F., Costa J.P.O., Silva A.R. (2019). The Role of the LY294002—A Non-Selective Inhibitor of Phosphatidylinositol 3-Kinase (PI3K) Pathway- in Cell Survival and Proliferation in Cell Line SCC-25. Asian Pac. J. Cancer Prev..

[82] Abdallah M.E., El-Readi M.Z., Althubiti M.A., Almaimani R.A., Ismail A.M., Idris S., Refaat B., Almalki W.H., Babakr A.T., Mukhtar M.H. (2020). Tamoxifen and the PI3K Inhibitor: LY294002 Synergistically Induce Apoptosis and Cell Cycle Arrest in Breast Cancer MCF-7 Cells. Molecules.

[83] Colaprico A., Silva T.C., Olsen C., Garofano L., Cava C., Garolini D., Sabedot T.S., Malta T.M., Pagnotta S.M., Castiglioni I. (2016). TCGAbiolinks: An R/Bioconductor package for integrative analysis of TCGA data. Nucleic Acids Res..

[84] Love M.I., Huber W., Anders S. (2014). Moderated estimation of fold change and dispersion for RNA-seq data with DESeq2. Genome Biol..

[85] Langfelder P., Horvath S. (2008). WGCNA: An R package for weighted correlation network analysis. BMC Bioinform..

[86] Guo K., Yang J., Jiang R., Ren X., Liu P., Wang W., Zhou S., Wang X., Ma L., Hu Y. (2024). Identification of Key Immune and Cell Cycle Modules and Prognostic Genes for Glioma Patients through Transcriptome Analysis. Pharmaceuticals.

[87] Zhang B., Horvath S. (2005). A general framework for weighted gene co-expression network analysis. Stat. Appl. Genet. Mol. Biol..

[88] Adebayo O.O., Dammer E.B., Dill C.D., Adebayo A.O., Oseni S.O., Griffen T.L., Ohandjo A.Q., Yan F., Jain S., Barwick B.G. (2022). Multivariant Transcriptome Analysis Identifies Modules and Hub Genes Associated with Poor Outcomes in Newly Diagnosed Multiple Myeloma Patients. Cancers.

[89] Robin X., Turck N., Hainard A., Tiberti N., Lisacek F., Sanchez J.-C., Müller M. (2011). pROC: An open-source package for R and S+ to analyze and compare ROC curves. BMC Bioinform..

[90] Yu G., Wang L.-G., Han Y., He Q.-Y. (2012). clusterProfiler: An R package for comparing biological themes among gene clusters. OMICS J. Integr. Biol..

