# Supplementary material for: Gene Co-Expression Network Analysis Associated with Endometrial Cancer Tumorigenesis and Survival Outcomes
Source: Int J Mol Sci. 2024 Nov 18;25(22):12356. doi: 10.3390/ijms252212356 (PMC11594472; doi:10.3390/ijms252212356)
Supplement: Supplementary file 1 [file ijms-25-12356-s001.zip › 20241104-Supplemental Files.pdf]

**SUPPLEMENTAL FILES**

**Supplemental Table 1. Clinical Data Demographics**

| | **Count** | **Category** |
|---|---|---|
| american indian or alaska native | 4 | Race |
| asian | 20 | Race |
| black or african american | 112 | Race |
| native hawaiian or other pacific islander | 9 | Race |
| not reported | 32 | Race |
| white | 399 | Race |
| NA | 12 | Race |
| 20-40 | 19 | Age_Range |
| 41-60 | 200 | Age_Range |
| 61-80 | 307 | Age_Range |
| 81+ | 47 | Age_Range |
| NA | 15 | Age_Range |

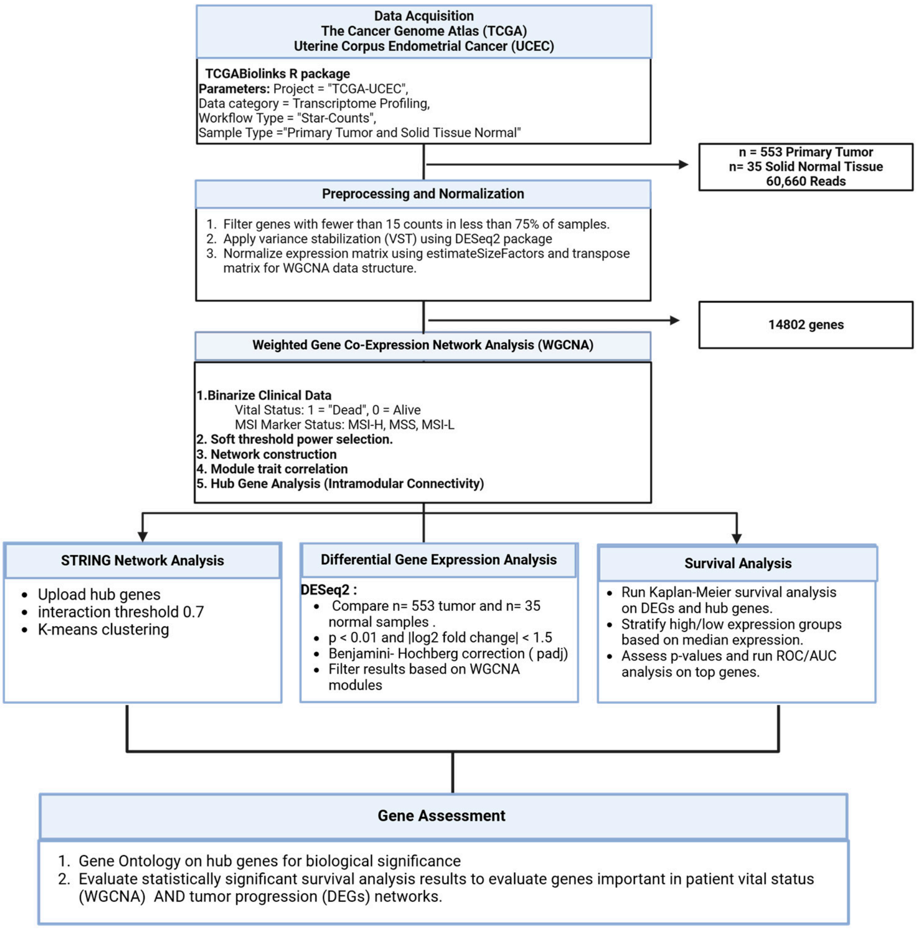


**Supplemental Figure 1.** Overview of methodology including data acquisition ins TCGABiolinks, Preprocessing and Normalization, WGCNA, STRING, Differential Gene Expression Analysis, and Survival analysis.
